# Clinical Staging Versus Biomarker-Guided Initiation of Continuous Renal Replacement Therapy: A Systematic Review and Meta-Analysis

**DOI:** 10.7759/cureus.103128

**Published:** 2026-02-06

**Authors:** Kevin Tran, Daniel Bach, George M Wilkins, Paramveer S Brar, Zachary Yamada, Talal El-Hefnawy

**Affiliations:** 1 College of Osteopathic Medicine, California Health Sciences University, Clovis, USA; 2 Biomedical Sciences, California Health Sciences University, Clovis, USA

**Keywords:** acute kidney injury, continuous renal replacement therapy, early versus delayed initiation, kdigo staging, mortality, ngal biomarker, timing of initiation

## Abstract

The most influential timing of continuous renal replacement therapy (CRRT) in gravely ill patients with acutely severe, uncompensated renal dysfunction continues to be a subject of ongoing debate. Although earlier initiation has been hypothesized to limit metabolic disturbances and prevent downstream organ dysfunction, clinical trials have yielded inconsistent findings, in part because of variability in how “early” initiation is defined across studies. This meta-analysis, in conjunction with a systematic review, sought to examine the correlation between early versus delayed CRRT initiation and mortality, with analyses stratified according to initiation strategy, including clinical staging-based benchmark by Kidney Disease: Improving Global Outcomes (KDIGO) and biomarker-driven approaches using neutrophil gelatinase-associated lipocalin (NGAL).

A literature search was conducted in PubMed, EMBASE, and the Cochrane Library to identify studies published between January 2015 and June 2025. Eligible studies included randomized controlled trials and observational investigations that compared early and delayed CRRT initiation among critically ill adults with acute kidney injury (AKI). The primary outcome of interest was all-cause mortality measured between 28 and 90 days or at the time of intensive care unit (ICU) discharge. Pooled relative risks (RR) and odds ratios (OR) were estimated using random-effects meta-analytic models, with subgroup analyses performed according to initiation criteria.

Nine studies encompassing 2,349 patients were included (six randomized trials and three observational studies). Overall, early CRRT initiation was not associated with a statistically significant decrease in mortality compared with delayed initiation (risk ratio (RR) = 0.87; 95% confidence interval (CI), 0.69-1.10; p = 0.25; I^2^ = 90.4%). Subgroup analysis demonstrated no significant mortality benefit with biomarker-guided (NGAL-based) early initiation (RR = 0.90; 95% CI, 0.41-2.01), whereas KDIGO-based initiation showed a borderline association favoring early therapy (RR = 0.75; 95% CI, 0.57-0.99), though heterogeneity remained substantial. No meaningful interaction was observed between initiation strategy and mortality.

## Introduction and background

Acute kidney injury (AKI) remains a frequent and devastating complication amongst critically ill patients, profoundly influencing mortality and long-term prognosis [1]. The global incidence of AKI in critically ill patients ranges from 30% to 60% [1], with one study reporting 90-day mortality rates to be 23% [2]. Among available modalities, continuous renal replacement therapy (CRRT) is indicated in unstable patients because its gradual, continuous solute and fluid removal is better tolerated than intermittent therapies [3].

Traditionally, the decision to initiate CRRT has been guided by overt physiological and biochemical derangements, such as refractory volume overload, life-threatening hyperkalemia, severe metabolic acidosis, or uremic symptoms [4]. Although some studies suggest that earlier initiation of CRRT during the course of renal dysfunction may offer clinical advantages, other evidence, particularly in septic shock, indicates that early therapy may not improve outcomes and could, in some circumstances, be harmful [5]. This uncertainty has fueled an ongoing debate in critical care nephrology regarding the optimal timing of CRRT to maximize survival and promote renal recovery. The timing of CRRT may influence outcomes because early therapy has the potential to interrupt worsening acidosis, fluid accumulation, and metabolic derangements before they contribute to secondary organ injury.

Several concepts of “early” initiation have been proposed, each aiming to intervene before organ dysfunction becomes irreversible. Within the current evidence, these concepts can be broadly classified into two predominant approaches. One approach is clinical staging-based initiation, which is typically guided by Kidney Disease: Improving Global Outcomes (KDIGO) stage 2-3 criteria [6]. The other approach is biomarker-driven initiation, which uses markers such as neutrophil gelatinase-associated lipocalin (NGAL) to detect early tubular injury before overt rises in serum creatinine [7,8].

NGAL has emerged as a promising early biomarker for AKI, capable of detecting tubular injury hours to days before creatinine rises and potentially enabling earlier, biologically guided initiation of CRRT [9]. However, its clinical utility remains uncertain, as evidence evaluating NGAL-guided timing remains limited. Growing interest in NGAL reflects an effort to incorporate more biologically informed markers into decisions about when to initiate kidney support therapies.

Nonetheless, NGAL represents only one of several strategies used to define early initiation, and differences among studies have led to a wide range of reported findings. Trials such as ELAIN demonstrated a mortality benefit with early initiation [10], whereas AKIKI, IDEAL-ICU, and STARRT-AKI found no survival advantage and, in some cases, increased treatment exposure without clinical benefit [11-13]. Much of the inconsistency across studies likely reflects differences in how "early" initiation is defined, with varying biomarker thresholds and KDIGO criteria contributing to methodological variation rather than true differences in treatment effect.

This meta-analysis aims to evaluate the impact of early versus delayed CRRT initiation using a stratified framework that distinguishes KDIGO-based and biomarker-guided (NGAL-based) definitions of early initiation. By incorporating these distinctions, this study seeks to determine whether the effectiveness of early CRRT initiation depends on the criteria used to trigger therapy and whether biomarker-guided strategies offer advantages over traditional creatinine-based approaches.

## Review

Methods

Search Design

This study was designed and reported in alignment with the Preferred Reporting Items for Systematic Reviews and Meta-Analyses (PRISMA) 2020 recommendations for systematic reviews and meta-analyses [14]. We examined whether the timing of CRRT initiation (early vs. delayed) influenced clinical outcomes in patients with AKI. Ethics approval and informed consent were not required, as this study analyzed data from previously published randomized and observational research. To allow for methodological rigor and reproducibility, all significant elements of the study protocol were organized and established prior to study initiation.

Population, Intervention, Comparison, Outcomes, and Study (PICOS) Framework

The research question was formulated using the PICOS framework to define the key components guiding this review [15]. The population comprised critically ill patients with AKI who required CRRT. The intervention of interest was early initiation of CRRT, as defined by study-specific criteria such as KDIGO stage 2-3 classification, biomarker-based thresholds including NGAL, or early clinical indicators. The comparator was delayed initiation of CRRT following additional renal or clinical deterioration. The primary outcome was all-cause mortality measured between 28 and 90 days or at intensive care unit (ICU) discharge. Secondary outcomes included renal recovery, dependence on CRRT, and length of stay in the ICU or hospital.

Search Strategy

The literature search was performed in accordance with the Preferred Reporting Items for Systematic Reviews and Meta-Analyses (PRISMA) 2020 recommendations to identify studies relevant to the objectives of this meta-analysis. Comprehensive searches were conducted across three distinct databases (PubMed, EMBASE, and the Cochrane Library) from January 2015 through June 2025. The PubMed search employed the following Medical Subject Headings (MeSH) terms and keywords: ("Acute Kidney Injury"[Mesh] OR "AKI") AND ("Continuous Renal Replacement Therapy"[Mesh] OR "CRRT" OR "continuous renal replacement therapy" OR "renal dialysis") AND ("Critical Illness"[Mesh] OR "critically ill" OR "ICU") AND ("Time Factors"[Mesh] OR "early initiation" OR "timing" OR "lipocalin-2"[Mesh] OR "biomarkers"[Mesh] OR "biomarker-guided" OR "KDIGO" OR "creatinine") AND ("Mortality"[Mesh] OR "Treatment Outcome"[Mesh] OR "renal recovery" OR "Recovery of Function"[Mesh]).

A similar search strategy, appropriately modified to align with indexing terms and controlled vocabulary specific to each database, was executed in EMBASE and the Cochrane Library. Searches were limited to English-language publications or studies with English-language data available.

Eligibility Criteria

Studies were considered eligible if they evaluated patients in critical conditions with AKI who received CRRT and compared various initiation strategies (early and delayed). Eligible study designs included randomized controlled trials (RCTs), retrospective cohort analyses, prospective cohort analyses, and pre-post intervention analyses. Studies were required to report at least one of the following outcomes: all-cause mortality, renal recovery, dependence on CRRT, or ICU/hospital length of stay. Only human studies published with English-accessible data between January 2015 and June 2025 were included. Exclusion criteria included studies evaluating intermittent or hybrid modalities, case reports, review articles, conference abstracts, editorials, and studies lacking comparative data between early and delayed CRRT initiation.

Study Selection

**Figure 1:**
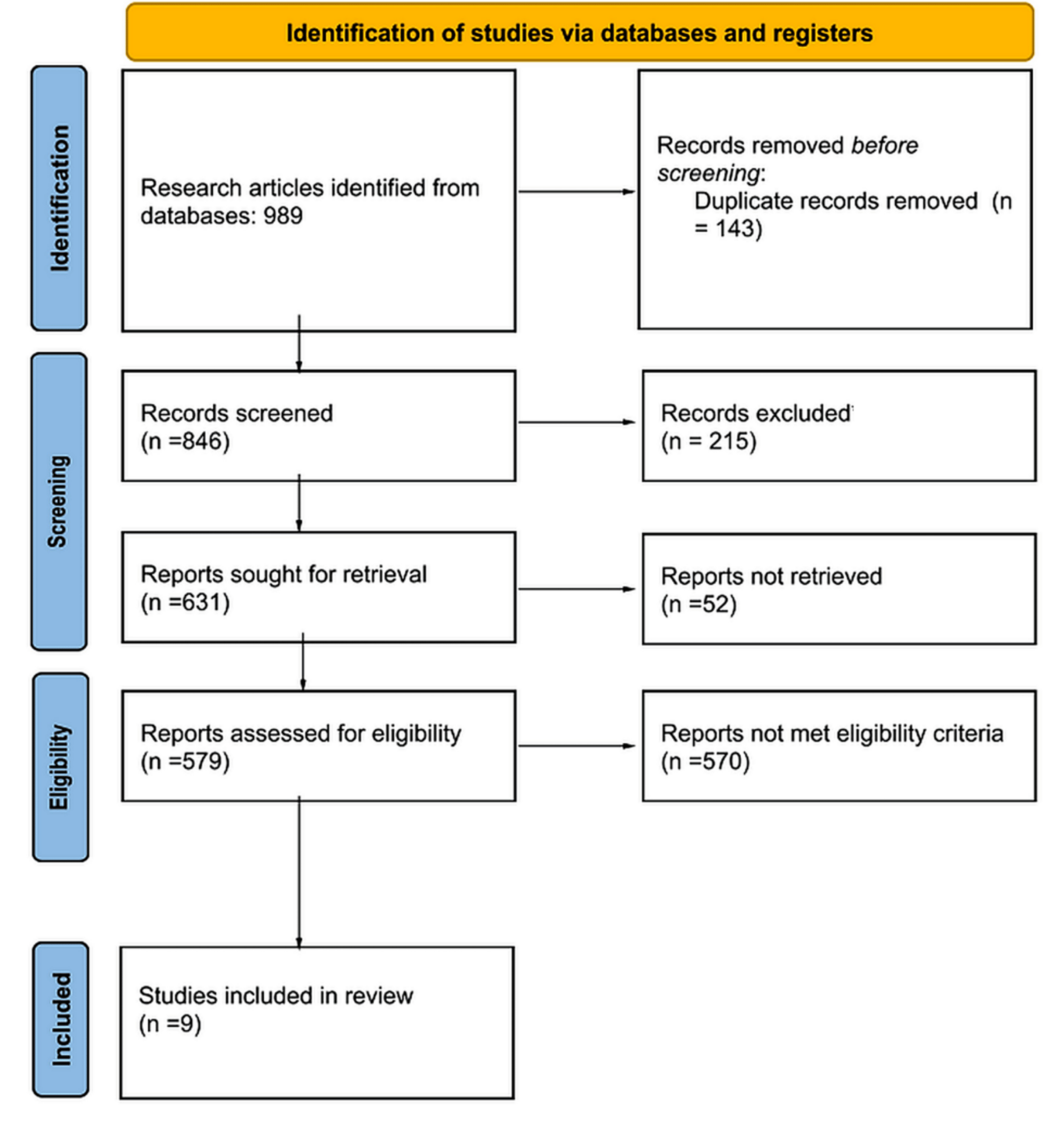
PRISMA flow chart for screening and selection of included studies PRISMA: Preferred Reporting Items for Systematic Reviews and Meta-Analyses

All retrieved citations were uploaded into Rayyan (Rayyan Systems Inc., Cambridge, MA, USA) for reference management and duplicate identification, with duplicates verified and removed prior to screening. Two reviewers independently evaluated abstracts based on pre-established eligibility criteria. Articles meeting initial screening criteria underwent a comprehensive evaluation to deduce final eligibility. Disagreements between reviewers were remedied through discussion, with involvement of additional reviewers when necessary to achieve consensus. The selection workflow followed PRISMA recommendations, and study counts at each screening phase are summarized in the PRISMA flow diagram (Figure 1).

Data Extraction

Data extraction followed a standardized methodology consistent with PRISMA guidance. Search results from all databases were imported into Rayyan for citation management, where duplicate records were identified using automated detection and verified prior to exclusion. Two reviewers independently extracted predefined variables from all eligible studies using a structured data abstraction form. Any discrepancies were resolved through discussion, with involvement of additional reviewers when consensus could not be reached.

Information collected included bibliographic details (first author and publication year), study design (RCTs, retrospective cohorts, or pre-post intervention studies), participant characteristics (age group and sample size), intervention definitions (criteria used to define early initiation), duration of follow-up, and outcome data for all-cause mortality. Extracted data were compiled into a centralized spreadsheet, and accuracy was ensured through independent cross-verification by reviewers.

Risk of Bias Assessment

Methodological rigor of RCTs was assessed with the Cochrane Risk of Bias 2 (ROB-2) framework, while non-randomized studies were assessed with the Risk Of Bias In Non-randomized Studies of Interventions (ROBINS-I) instrument [16,17]. Studies were subsequently categorized as having low, moderate, or serious overall risk of bias.

Statistical Analysis

Analysis of the data via statistical tests was performed using IBM SPSS Statistics for Windows, Version 31 (Released 2025; IBM Corp., Armonk, New York, United States). Mortality outcomes were obtained from all eligible studies, and effect sizes were displayed as relative risks (RRs)/odds ratios (ORs) with standard confidence intervals (95% CIs). Because variability across studies was anticipated in advance, pooled estimates were calculated using a pre-specified random-effects modeling approach. A meaningful association was noted if a probability value was less than or equal to 0.05.

Variability between studies was quantified using the I^2^ statistic, with values greater than 50% interpreted as representing substantial heterogeneity. To examine the influence of study design on the overall results, sensitivity analyses were performed by removing the retrospective cohort and pre-post intervention studies, thereby restricting analyses to RCTs. Forest plots were generated for both RR and OR estimates, and subgroup analyses were conducted according to initiation strategy, comparing NGAL-guided and KDIGO-guided approaches.

Results

Study Selection

The literature search yielded 989 records across databases. Following automated and manual deduplication, 143 records were removed, leaving 846 citations for initial screening, of which 215 were excluded based on titles and abstracts. Retrieval was attempted for 631 reports; 52 could not be accessed. Full-text evaluation was completed for 579 reports, with 570 excluded after eligibility assessment. The final dataset included six RCTs, one retrospective cohort study, one prospective cohort study, and one pre/post intervention study [11-13,18-23]. This process is explicitly detailed in Figure 1.

Table 1 summarizes the key characteristics of the studies included in this meta-analysis, including study design, sample size, definitions of early versus late CRRT initiation, diagnostic criteria for renal replacement therapy, mortality outcomes, and follow-up duration.

**Table 1:** Characteristics of included studies evaluating early versus delayed CRRT initiation This table summarizes study design, patient group allocation, diagnostic criteria used to define early versus delayed initiation of CRRT, mortality outcomes, and follow-up duration across included studies. Early and late groups reflect study-specific timing criteria based on clinical staging, laboratory thresholds, or biomarker guidance. AKI: acute kidney injury; BUN: blood urea nitrogen; CRRT: continuous renal replacement therapy; E: early initiation group; ICU: intensive care unit; KDIGO: Kidney Disease: Improving Global Outcomes; L: delayed initiation group; NGAL: neutrophil gelatinase-associated lipocalin; PaO_2_/FiO_2_: ratio of arterial oxygen partial pressure to fractional inspired oxygen; pNGAL: plasma neutrophil gelatinase-associated lipocalin; RAI: Renal Angina Index; RCT: randomized controlled trial; RIFLE: Risk, Injury, Failure, Loss, End-stage kidney disease criteria; RRT: renal replacement therapy; TF: treatment framework; uNGAL: urinary neutrophil gelatinase-associated lipocalin; UO: urine output

Author, Year	Study Design	Study Groups	Criteria for Early RRT	Criteria for Late RRT	Mortality	Follow-Up
Zarbock et al., 2016 (ELAIN) [11]	RCT	E: 112 L: 119	Within eight hours after a KDIGO Stage 2 diagnosis	KDIGO Stage 3 + (UO <200 mL/day, anuria, organ edema), or marked metabolic derangement (urea >100 mg/dL, or K⁺ >6 mmol/L, or Mg2⁺ >4 mmol/L)	E:44/112 L:65/119	90 days
Gaudry et al., 2016 (AKIKI) [12]	RCT	E: 312 L: 308	Within six hours after a KDIGO Stage 3 diagnosis	Pulmonary edema, prolonged oliguria (>72 hours), or marked metabolic disturbances, including elevated BUN (>40 mmol/L), metabolic acidosis (pH <7.15), and marked hyperkalemia (K⁺ >5.5 mmol/L).	E: 150/311 L: 153/308	60 days
Barbar et al., 2018 (IDEAL-ICU) [13]	RCT	E: 246 L: 238	Within 12 hours after the RIFLE-F (Failure) stage diagnosis	Pulmonary edema, marked hyperkalemia (K⁺ >6.5 mmol/L), or marked metabolic acidosis (pH <7.20)	E: 138/239 L: 128/238	90 days
Bagshaw et al., 2020 (STARRT-AKI) [18]	RCT	E: 1465 L: 1462	KDIGO Stage 2 or 3	Lack of renal recovery ≥72 hours after randomization; volume overload with PaO_2_/FiO_2_ ≤200; marked acid-base disturbance (pH ≤7.20 or HCO_3_⁻ ≤12 mmol/L); or marked hyperkalemia (K⁺ ≥6.0 mmol/L)	E: 643/1489 L: 639/903	90 days
Xia et al., 2019 [19]	Prospective cohort	E: 30 L: 30	uNGAL > 1310 ng/mL	Pulmonary edema, marked hyperkalemia (K⁺ >6.5 mmol/L), or marked metabolic acidosis (pH <7.20)	E: 15/30 L: 15/30	28 days
Wald et al., 2015 [20]	RCT	E: 48 L: 52	Creatinine ≥ 354 µmol/L, or ≥2× baseline creatinine, or UO <6 mL/kg/12 hours	Marked hyperkalemia (K⁺ >6.0 mmol/L), marked metabolic acidosis reflected by HCO_3_⁻ <10 mmol/L, or hypoxemia associated with pulmonary edema (PaO_2_/FiO_2_ <200).	E: 18 L: 19	90 days
Srisawat et al., 2018 [21]	Feasibility RCT	E: 20 L: 40	pNGAL > 400 ng/mL; Within 12 hours of randomization	Elevated plasma NGAL (>400 ng/mL) in combination with at least one severe clinical criterion, including elevated BUN (>22 mmol/L), hyperkalemia (>6.2 mmol/L), metabolic acidosis (pH <7.20 or HCO_3_⁻ <15 mEq/L), persistent oliguria or anuria, or profound edema.	E: 3/20 L: 19/40	28 days
Goldstein et al., 2023 [22]	Pre-post intervention study	Pre-TF2: 71 Post-TF2: 107	Post-TF2: Renal Angina Index (RAI) ≥ 8 + NGAL ≥ 150 ng/mL	Pre-TF2: based on clinician discretion	Pre-TF2: 38/71 Post-TF2: 37/107	Until ICU discharge
Xing et al., 2019 [23]	Retrospective cohort	E: 57 L: 84	Within 12 hours after the RIFLE-F (Failure) stage diagnosis	Oliguria (UO <0.3 mL/kg/h for ≥24 hours), anuria ≥12 hours, or creatinine 3x baseline (or ≥350 μmol/L) with rapid increase ≥44 μmol/L	E: 9/57 L: 22/84	28 days

Characteristics of Included Studies

This meta-analysis comprised six trials in a randomized controlled setting in addition to three trials taking the form of retrospective, prospective, and pre/post intervention studies, totaling 2,349 patients with AKI [11-13,18-23]. The primary objective across studies was to evaluate the impact of early versus delayed CRRT initiation in critically ill patients. Key study characteristics are summarized in Table 1. Sample sizes ranged from 20 to 1,465 participants, and follow-up durations ranged from 28 to 90 days, with one study following patients until ICU discharge. Analyses were conducted using a random-effects model to take into account anticipated inter-study fluctuations.

Risk of Bias Assessment

**Figure 2:**
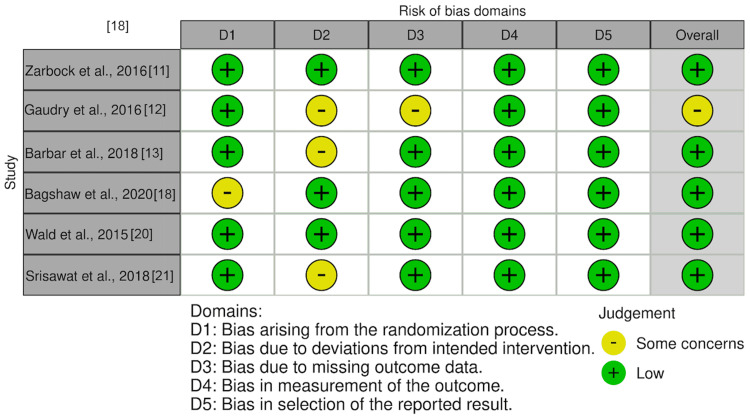
Risk of bias from randomized studies Studies included [11-13,18,20,21]

**Figure 3:**
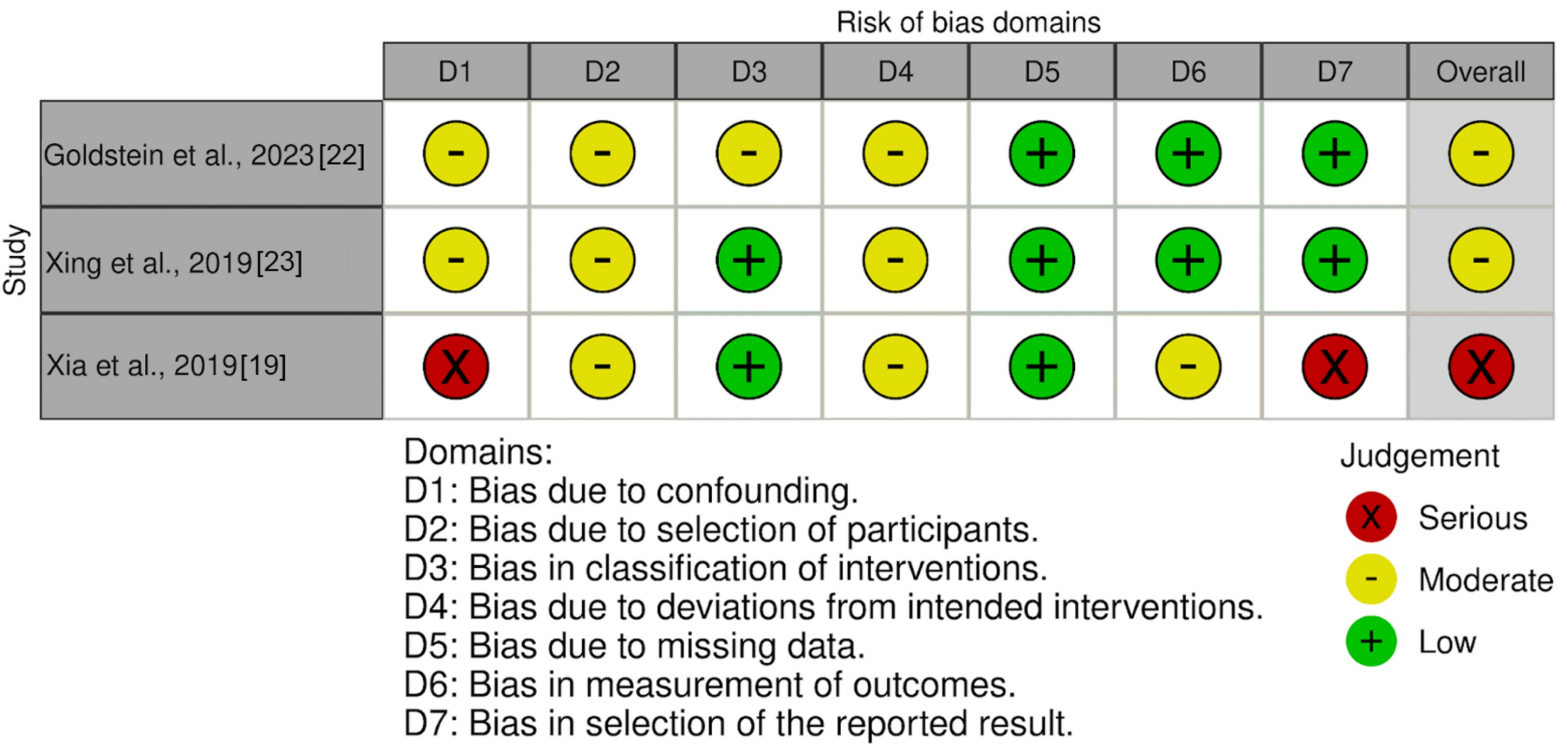
Risk of bias from non-randomized studies Studies included [22,23,19]

The quality of the collected studies was evaluated, independently, by two individual reviewers using standard tools to determine the risk of bias. In the case of RCTs, the Cochrane ROB-2 (Figure 2) [16]. Non-randomized studies, including retrospective and prospective cohort studies as well as pre-post intervention designs, were appraised using the ROBINS-I tool (Figure 3) [17]. Conflicts in assessment were remedied by input from a third reviewer.

Overall, most RCTs demonstrated low to some concerns about risk of bias, with isolated issues related to missing data or deviations from intended interventions. In a retrospective view, trials lacking randomization presented with an intermediate to significant risk of bias, predominantly driven by common and unknown variables that are shared between participants and challenges inherent to observational study designs. Many of these studies were retrospective, limiting the ability to control for baseline differences, co-morbidities, and missing data, factors that contributed to a higher risk of bias across multiple ROBINS-I domains.

Primary Outcomes

**Figure 4:**
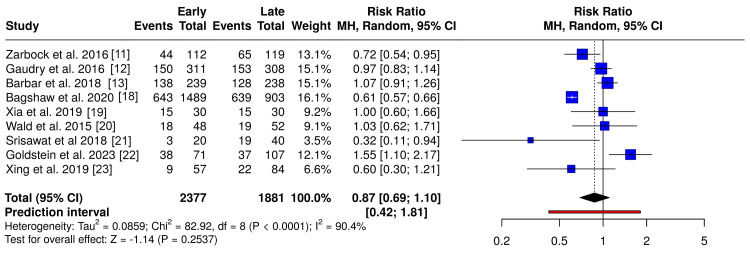
Overall mortality comparing early versus delayed CRRT initiation (relative risk) Forest plot comparing early versus delayed CRRT initiation for all-cause mortality using relative risks. Random-effects models were used. CRRT: continuous renal replacement therapy Studies included [11-13,18-23]

**Figure 5:**
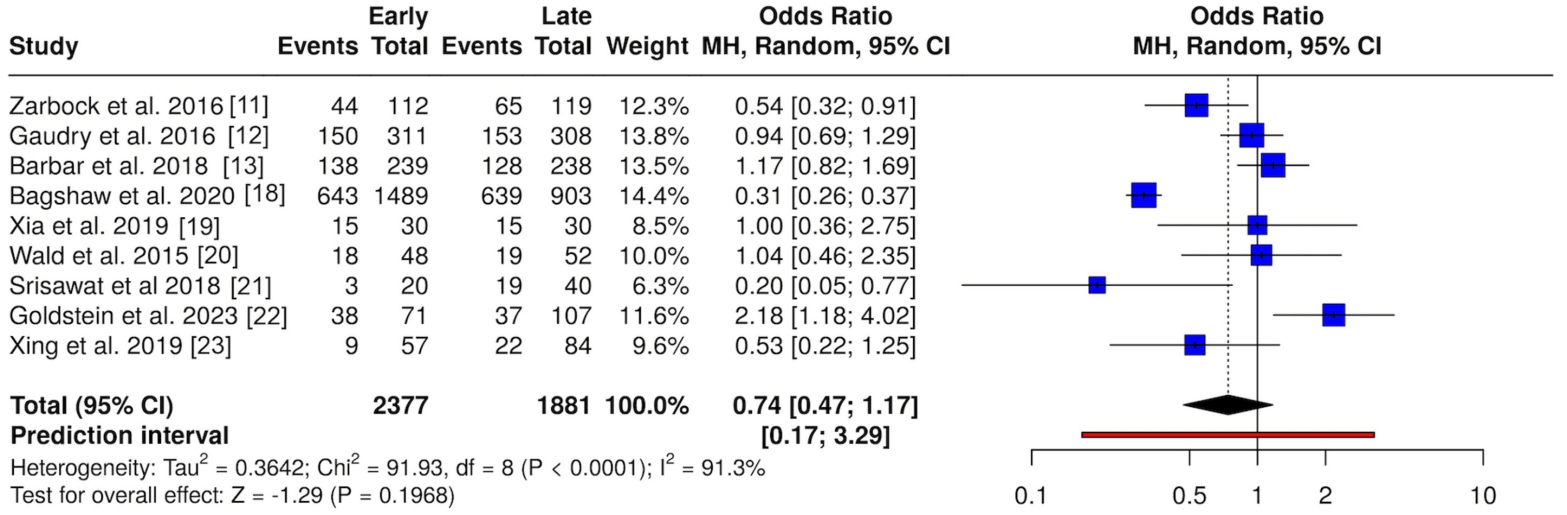
Overall mortality comparing early versus delayed CRRT initiation (odds ratio) Forest plot comparing early versus delayed CRRT initiation for all-cause mortality using odds ratios. Random-effects models were used. CRRT: continuous renal replacement therapy Studies included [11-13,18-23]

After applying inclusion and exclusion criteria, nine studies were included in the pooled analysis of mortality outcomes comparing early versus late CRRT initiation. All studies reported mortality events and total participants within the early and delayed CRRT groups. Using a random-effects model, the pooled RR displayed no underlying significant change in the statistical outcome difference in risk of death between different initiation times (RR = 0.87; 95% CI, 0.69-1.10; p = 0.25), with substantial heterogeneity (I^2^ = 90.4%) (Figure 4). A complementary analysis using OR produced consistent findings (OR = 0.74; 95% CI, 0.47-1.17; p = 0.20) (Figure 5).

**Figure 6:**
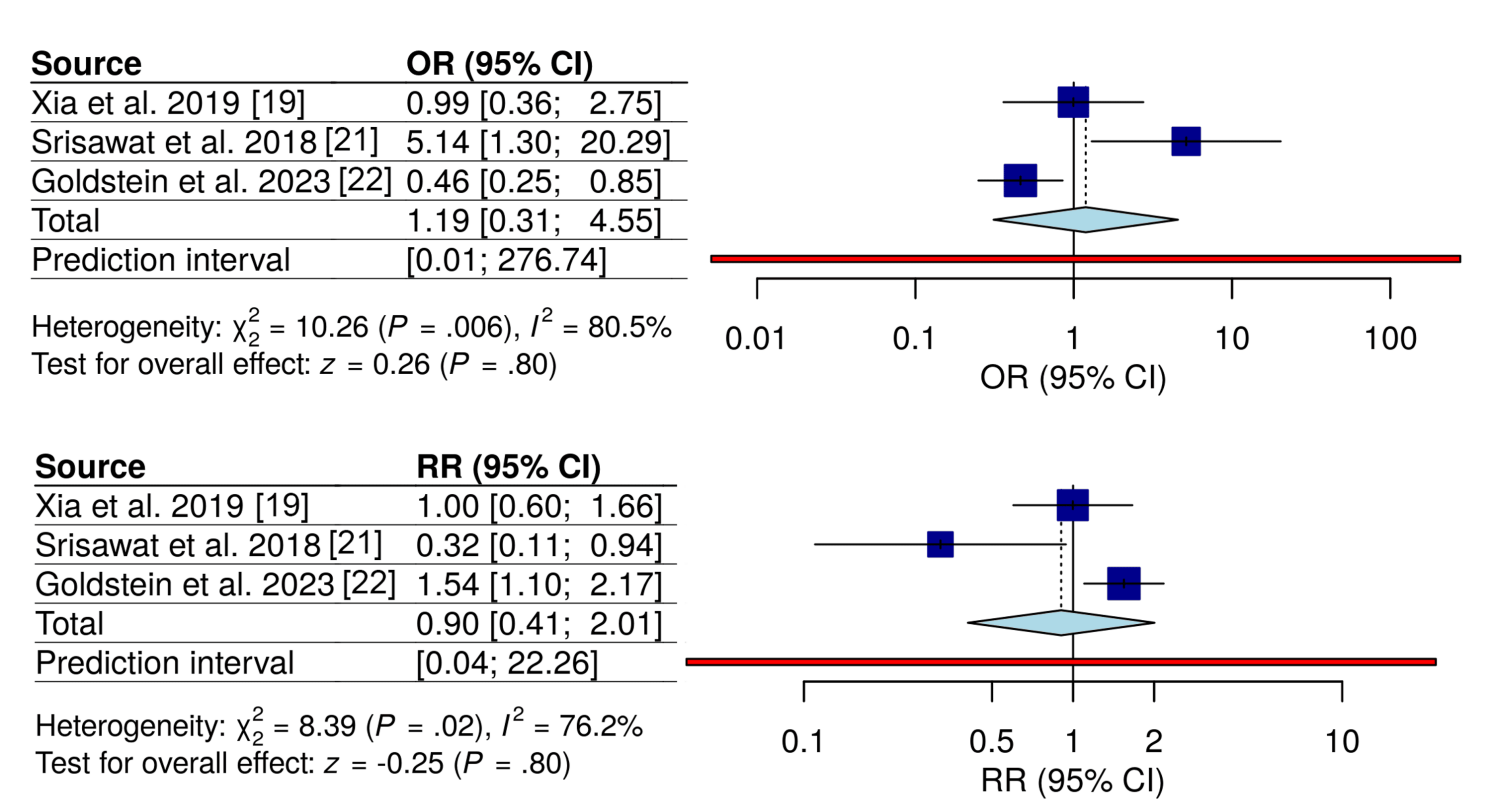
Subgroup analysis of mortality by NGAL-guided CRRT initiation Forest plots comparing early versus delayed CRRT initiation using NGAL-based criteria. Odds ratios and relative risks are shown. Random-effects models were used. NGAL: neutrophil gelatinase-associated lipocalin; CRRT: continuous renal replacement therapy Studies included [19,21,22]

To evaluate whether the definition of early CRRT initiation influenced mortality outcomes, subgroup analyses were stratified according to primary initiation criteria, including biomarker-guided (NGAL-based) and clinically defined (KDIGO-based) approaches. In the NGAL-guided subgroup, early initiation was not associated with a significant reduction in mortality, whether assessed using RR (RR = 0.90; 95% CI, 0.41-2.01) or OR (OR = 1.19; 95% CI, 0.31-4.55), with wide CIs and substantial heterogeneity (I^2^ = 76-81%) (Figure 6).

**Figure 7:**
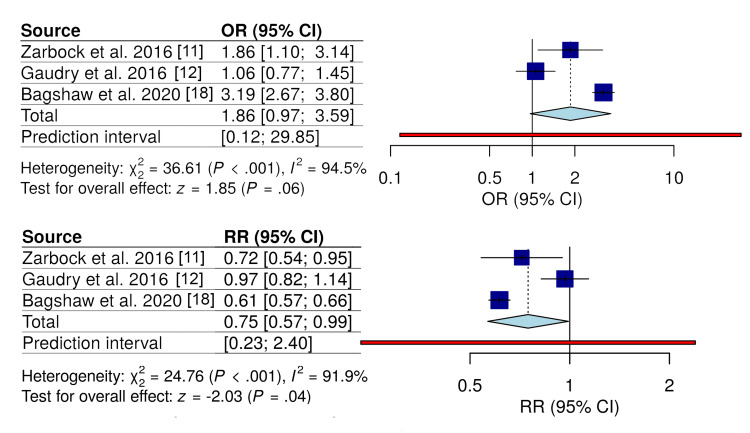
Subgroup analysis of mortality by KDIGO-guided CRRT initiation Forest plots comparing early versus delayed CRRT initiation using KDIGO-based criteria. Odds ratios and relative risks are shown. Random-effects models were used. KDIGO: Kidney Disease: Improving Global Outcomes; CRRT: continuous renal replacement therapy Studies included [11,12,18]

In contrast, the KDIGO-based subgroup demonstrated a more consistent, borderline association favoring early initiation (RR = 0.75; 95% CI, 0.57-0.99; OR = 1.86; 95% CI, 0.97-3.59), although heterogeneity remained high (I^2^ ≈ 92%) (Figure 7).

Overall, no statistically meaningful interaction was observed between initiation criteria and mortality outcomes. Although both biomarker-guided and clinically guided strategies demonstrated similar directional trends, the wide CIs and persistent heterogeneity suggest that differences in initiation criteria, patient selection, and baseline illness severity likely account for the observed variation rather than reflecting a consistent treatment effect.

Discussion

This review synthesized evidence from nine studies, including six RCTs, one prospective cohort study, one retrospective cohort study, and one pre/post intervention study. Across 2,349 patients, pooled analyses using both RR and OR models demonstrated no statistically significant difference in all-cause mortality between early and delayed initiation strategies, although both effect estimates trended toward lower mortality with earlier initiation. Subgroup analyses stratified by initiation criteria, biomarker-guided (NGAL-based) versus clinical staging-based (KDIGO), yielded consistent findings, with neither approach demonstrating a significant mortality benefit.

Collectively, these results suggest that while early CRRT initiation may confer physiological advantages in selected patients, its effect on survival likely depends on individual patient characteristics, underlying illness severity, and the criteria used to trigger therapy rather than the timing of initiation alone.

Interpretation of Findings

The lack of a statistically significant mortality benefit underscores the complex interplay between systemic illness, renal pathophysiology, and the timing of renal support. Early CRRT is theoretically advantageous for mitigating complications of severe AKI, including metabolic acidosis, electrolyte imbalance, and fluid overload, thereby stabilizing organ function and reducing secondary injury [18]. Proposed mechanistic benefits of earlier intervention also include enhanced clearance of pro-inflammatory mediators and modulation of cytokine burden [11].

However, CRRT is not without risk, as early initiation may expose patients to unnecessary extracorporeal circulation and anticoagulation-related complications, while the therapy itself carries procedural risks associated with catheter placement and extracorporeal circuitry [3]. Earlier therapy may also lead to greater treatment exposure overall, which can increase logistical demands and procedural complexity without guaranteeing clinical benefit.

Although not statistically significant, the trend toward lower mortality with earlier initiation could still represent a clinically relevant signal in specific high-risk populations. In high-risk subgroups such as patients with septic shock, multiorgan dysfunction, or refractory fluid overload, early initiation may interrupt the trajectory of metabolic derangement and prevent irreversible organ injury [24]. Conversely, in less severe or hemodynamically stable cases, a delayed or watchful-waiting strategy may prevent unnecessary intervention without compromising outcomes [25]. Collectively, these findings support individualized timing guided by dynamic assessment of hemodynamics, urine output, and biochemical trends, rather than a uniform early initiation strategy for all patients [26].

Comparison With Previous Evidence

The present findings align with several landmark RCTs and prior meta-analyses demonstrating no definitive survival benefit with early initiation of CRRT. Large multicenter trials such as AKIKI, IDEAL-ICU, and STARRT-AKI consistently reported similar mortality rates between early and delayed initiation strategies, although early groups experienced longer exposure to renal replacement therapy [11-13]. In AKIKI, nearly half of patients assigned to the delayed arm never required CRRT, highlighting that delayed strategies can safely avoid unnecessary intervention in a substantial proportion of patients [12]. STARRT-AKI, the largest randomized trial to date, likewise demonstrated no improvement in 90-day mortality with early initiation and reported increased dialysis dependence among survivors treated early [18].

Conversely, smaller studies, including the single-center ELAIN trial and observational work by Xing et al., identified reductions in mortality associated with earlier initiation [11,23]. These discrepancies likely reflect differences in patient selection, illness severity, and operational definitions of early initiation. ELAIN, for example, initiated CRRT at KDIGO stage 2, which was substantially earlier than most large multicenter trials, and enrolled a relatively homogeneous surgical ICU population, factors that may have influenced observed outcomes [11]. Taken collectively, prior evidence suggests that the effect of timing is highly context-dependent and strongly influenced by heterogeneity in study design and initiation thresholds.

This meta-analysis uniquely contributes by explicitly stratifying studies according to their initiation definitions, an approach not employed in prior landmark trials and reviews that predominantly evaluated KDIGO- or creatinine-based criteria. Using this stratified framework, mortality outcomes did not differ significantly between biomarker-guided and KDIGO-based initiation paradigms, suggesting that inconsistencies in how early initiation is defined account for much of the variability in reported outcomes. Biomarker-based strategies such as NGAL may nevertheless represent a promising frontier because they allow detection of subclinical tubular injury prior to overt biochemical deterioration. However, the limited number of NGAL-guided studies currently available restricts robust inference and underscores the need for further investigation.

Clinical Implications

From a clinical standpoint, these findings reinforce that a uniform early-initiation protocol is not universally appropriate for all critically ill patients with AKI. Timing should be individualized, integrating hemodynamic stability, degree of metabolic derangement, and underlying disease trajectory. In patients with refractory fluid overload, worsening acidemia, or progressive AKI despite optimized medical therapy, earlier CRRT initiation may still confer benefit. Conversely, stable patients without life-threatening complications can be safely observed, as demonstrated by the AKIKI trial in which nearly half of delayed-strategy patients never required CRRT [12].

Furthermore, this meta-analysis underscores the need for standardized and biologically relevant criteria for CRRT initiation. While KDIGO provides robust staging for AKI severity, it offers limited guidance on the transition from conservative management to renal support. Incorporating dynamic biomarkers such as NGAL, TIMP-2·IGFBP7, or cystatin C may refine decision-making by distinguishing patients with reversible injury from those progressing toward irreversible renal failure [27-29]. Integration of biomarker thresholds into clinical decision algorithms could also improve trial comparability and enhance predictive precision.

In practice, CRRT initiation should not rely solely on static biochemical thresholds or fixed time intervals. Clinicians should weigh the trajectory of illness, evaluating renal function trends, perfusion adequacy, and cumulative fluid balance. The findings of this review support an adaptive, physiology-guided strategy that emphasizes clinical context over predefined timing.

Strengths and Limitations

This meta-analysis has several key strengths. It is among the few to include both randomized and observational studies, allowing a more comprehensive assessment of CRRT timing in real-world intensive care populations. The use of a random-effects model accounted for clinical and methodological heterogeneity, and sensitivity analyses excluding non-RCTs confirmed the consistency and robustness of the main findings. Furthermore, stratification of studies based on initiation criteria (biomarker-guided versus KDIGO-based) provided a more nuanced interpretation of the timing debate, addressing a major limitation of prior pooled analyses that treated “early” initiation as a uniform construct.

However, several limitations must be acknowledged. Significant heterogeneity (I^2^ > 80%) was present across included studies, reflecting differences in patient populations, CRRT modalities, dosing strategies, and operational definitions of early versus delayed initiation. The inclusion of non-randomized studies, while enhancing generalizability, introduces potential confounding and selection bias, as patients with greater illness severity were more likely to receive earlier therapy. Additionally, inconsistencies in reporting secondary outcomes such as renal recovery, dialysis dependence, and length of stay limited the ability to perform pooled quantitative analyses. The relatively small number of eligible studies and predominance of single-center trials also constrain external validity. Finally, the possibility of publication bias cannot be excluded, as funnel plot and Egger’s test assessments were limited by the small number of studies available.

Future Directions

Future studies should redefine how AKI is detected and staged, moving beyond serum creatinine, which rises only after substantial nephron injury and may take 24-36 hours to increase following an insult due to slowed creatinine kinetics as GFR declines. Serum creatinine is also heavily influenced by age, muscle mass, and fluid status, all of which can dilute or obscure true renal dysfunction. Because of this delay and susceptibility to non-renal factors, creatinine frequently underestimates early kidney injury, masking “subclinical AKI” and narrowing the window in which interventions, such as optimal timing of CRRT, could be most effective.

Emerging biomarkers of kidney stress and tubular injury, such as NGAL, tissue inhibitor of metalloproteinase-2, insulin-like growth factor-binding protein 7, and cystatin C, can identify high-risk patients earlier and more accurately. Integrating these biomarkers with physiologic and hemodynamic parameters into standardized decision frameworks could refine patient selection, ensuring timely intervention while avoiding unnecessary therapy. Future multicenter trials should use biomarker-enriched enrollment, stratify by AKI etiology and illness severity, and adopt consistent reporting of outcomes such as mortality, renal recovery, and dialysis dependence. Economic and resource analyses will also be crucial in guiding practice, particularly in resource-limited critical care settings. Developing large collaborative registries and adaptive platform trials will further accelerate evidence generation and support precision-based, biology-driven timing strategies to improve patient outcomes.

## Conclusions

Variation in CRRT initiation timing was not associated with risk of death among critically ill patients with acute renal injury. Although early initiation consistently trended toward improved outcomes, substantial heterogeneity in initiation criteria, patient selection, and study methodology limits definitive conclusions. These findings support a personalized, physiology-driven approach to CRRT initiation rather than reliance on rigid temporal thresholds. Future work should prioritize the development of standardized, biomarker-integrated initiation criteria to determine whether targeted early intervention can meaningfully improve survival or renal recovery.
